# Relevance Study on Cerebral Infarction and Resistin Gene Polymorphism in Chinese Han Population

**DOI:** 10.14336/AD.2016.0201

**Published:** 2016-10-01

**Authors:** Aijuan Yan, Gaoyu Cai, Ningzhen Fu, Yulan Feng, Jialan Sun, Yiming Maimaiti, Weijun Zhou, Yi Fu

**Affiliations:** ^1^Department of Neurology & Institute of Neurology, and; ^2^Emergency Department, Rui Jin Hospital, School of Medicine, Shanghai Jiao Tong University, Shanghai 200025, China; ^3^Department of Neurology, Minhang Central Hospital, Shanghai 201100, China; ^4^Department of Neurology, Gongli Hospital, Shanghai 200135, China; ^5^Department of Neurology, the Second People’s Hospital of Kashgar, Kashgar 844000, China

**Keywords:** Cerebral infarction, Resistin, Single nucleotide polymorphism, Male, Small artery occlusion type

## Abstract

Recent research on genome-wide associations has implicated that the serum resistin level and its gene polymorphism are associated with cerebral infarction (CI) morbidity and prognosis, and could thereby regulate CI. This study aimed to investigate the association between the resistin single nucleotide polymorphism (SNP) and the susceptibility to CI in the Chinese Han population. A total of 550 CI patients and 313 healthy controls were genotyped. Nine SNPs of the resistin gene previously shown were sequenced and assessed for an association with CI. The numbers of GG genotype carriers of rs3219175 and rs3486119 in the CI group were significantly higher than those in the control group among the middle-aged group (aged 45-65), at 76% vs 67.9% (*P*=0.025) and 75.5% vs 67.9% (*P*=0.031). rs3219175 and rs34861192 were associated with CI in the dominant and superdominant models according to the genetic model analysis (*P*<0.05). Meanwhile, there was strong linkage disequilibrium among the rs34124816, rs3219175, rs34861192, rs1862513, rs3745367, 180C/G and rs3745369 sites. In a haplotype analysis, the occurrence rate of the haplotype AGGCAGC was 1.97 times (*P*<0.05) higher in the patient group than in the control group. In addition, the numbers of GG genotype carriers of rs3219175 and rs3486119 in the middle-aged male CI patients and the middle-aged small artery occlusion (SAO) CI patients were higher than those in the control group (*P*<0.05). In the Chinese Han middle-aged population, the GG gene type carriers of the resistin gene sites rs3219175 and rs34861192 had a high risk for CI onset, especially in middle-aged male patients and SAO CI in all middle-aged patients.

Cerebral infarction (CI), one of the main causes of death and disability in adults, increasingly threatens human health with its decreasing age of onset [1]. In China, deaths caused by CI is two thirds of the total number of deaths caused by stroke [2]. It has been implied that CI is a complicated clinical syndrome with diverse etiologies. Although it has been discovered that factors such as hypertension, diabetes, smoking and hyperlipoidemia play roles as primary risk factors for CI, many of the other factors leading to the disease, which could be a synergistic reaction of genetic and environmental factors, are still unclear [3-5]. Moreover, genetic variance is one of the main causes of CI [6]. It has been shown that genetic factors increase susceptibility to CI, though the exact genetic predisposition remains unclear [7]. Some studies have shown that gene mutations can affect common risk factors of CI, and further influence the risk, infarct size and prognosis of CI [8]. According to the evaluation of CI-related single nucleotide polymorphism (SNP), the total heritability of CI is approximately 37.9% [9]. Therefore, studies on genetic backgrounds have attracted increasing attention as an important part of CI-related research.

Resistin, a peptide hormones, was discovered and recognized by Steppan in studies on the mechanism of a new-type diabetes drug [10]. It possesses the characteristics of inflammation factors, such as inhibiting generation of adipose cells, enhancing resistance against insulin and regulating glycometabolism, which could finally lead to atherosclerosis [11]. RETN, located in the 13.3 area on the long arm of the 9 chromosome [12], is the resistin coding gene. Several SNPs have been discovered in RETN promoters, introns and 3′UTR (un-translated region) regions. It has also been shown that the gene polymorphism of RETN is related to obesity, type II diabetes, angiocardiopathy and non-alcohol fatty liver disease [13-16].

In a study on genes of 349 Japanese type II diabetes patients with CI complications, Tsukahara found that the rs1862513 allele on the resistin gene could be a risk marker of type II diabetes with CI [17]. Investigating Japanese residents over age 40, Osawa discovered not only a positive correlation between the serum resistin level and the occurrence of angiocardiopathy, but an increase in the rate of ischemic accidents induced by an increasing resistin level as well [18]. The results of Tsukahara and Osawa were also supported by other research, such as the finding of a higher risk for CI in patients with high resistin level and the GG gene type carriers of site rs1862513 announced by Nakashima [19]. Targeting CI patients, the resistin level influenced not only their disability rates but also their mortality [20]. In addition, resistin can be synthesized in the cerebral cortex and the hypothalamus [21]. Meanwhile, the cortical resistin mRNA level increased in the animal brain ischemia and brain injury models [22, 23], which thereby reminds us that there is a relationship between CI incidence and serum resistin level together with its gene polymorphism. The serum resistin level and its gene polymorphism could thus be one of the risk factors for CI.

However, no exact report about the direct relationship between CI and Chinese Han resistin gene polymorphism has been published until now. With the polymerase chain reaction-ligase detection reaction (PCR-LDR) technique [24], we selected 9 resistin gene sites (rs3745368, rs34124816, rs3219175, rs3219177, rs34861192, rs1862513, rs3745367, 180C/G and rs3745369) certified by the recent genome-wide association studies(GWAS)[25] and studied them in relation to the non-cardiac CI risk to examine whether they can be the basis of CI paroxysm in the Chinese Han population.

## MATERIALS & METHODS

### Subjects

Our target group consisted of hospitalized patients in the department of neurology at Rui Jin Hospital, Minhang Hospital and Gongli Hospital. There were a total of 550 patients (337 male, 213 female) between 45 and 75 years old, with the average age of 60±7 years. The diagnostic criteria for all patients can be found in the Guidelines for the diagnosis and treatment of cerebral infarction in China published in 2010[26]. With guidance from Trial of Org 10172 in Acute Stroke Treatment (TOAST) [27], all the patients were classified into two subtypes as either large artery atherosclerosis (LAA) CI or SAO CI. Inclusion criteria: (1) LAA CI and SAO CI (2) aged between 45-75 years old. Exclusion criteria: (1) cardiac embolism (CE), stroke of other demonstrated etiology (SDE) and stroke of other undemonstrated etiology (SUE) ; (2) coronary disease, peripheral vascular disease, therioma, severe liver and kidney function decline and other metabolic disorders such as thyroid dysfunction; (3) existence of family heredity history of CI; (4) age of less than 45 or more than 75 years.

313 control subjects (151 male, 162 female) consisted of physically healthy people examined in the same hospitals during the same period who were between 45 and 75 years old, with an average age of 58 ± 7. All the participants were part of the Han population living in Shanghai, China and did not include any sibling relationships. On the one hand, genetic factors impact the middle-aged population more significantly, according to previous research [28, 29]. On the other hand, it is necessary to lower the influence of uncontrolled factors to analyze the functional differences of genetic factors targeting different populations that have been divided according to their ages. Thus we divided the samples into a middle-aged group (45-65) and an elder group (>65) in light of the dividing criteria of the World Health Organization (WHO) [30, 31]. In our study, 634 middle-aged population (45-65) was consisted of 366 (226 male, 140 female) CI patients and 268 healthy control subjects (131 male, 137 female), while 229 elder people (66-75) were consisted of 184 CI patients (111 male, 73 female) and 45 healthy control subjects (20 male, 25 female). This study was approved by the ethics committee of Rui Jin Hospital that is affiliated with Shanghai Jiao Tong University School of Medicine.

### Methods

All the subjects fasted overnight for 12 hours before 5 ml fasting blood samples were collected in the morning. After an even mixture of the samples in an EDTA anticoagulation tube, 2 ml of the samples was prepared for DNA extraction while the other 3 ml was prepared for the test of serum glucose, triglyceride (TG), total cholesterol (TC), high-density lipoprotein cholesterol (HDL-C) and low-density lipoprotein cholesterol (LDL-C). All blood pressure statuses and complete clinical data were recorded.

A phenol-chloroform method was utilized to collect the blood whole genome DNA. Primer design software such as Primer Express and Oligo were applied for primer design (Table 1). A PCR-LDR test was then used to classify the SNPs (due to the little research on 180C/G that has been conducted, no corresponding primer name was found).

**Table 1:** Primer name, primer sequence and PCR length of the 9 sites

Primer name	Sequence (5’-3’)		PCR length (bp)
rs3745368	CGGCTCCAGGTCCGGAGG	TCAGATCCGCTCCATCATCA	111
rs34124816	TACAAGTGCTTGTCCGCACC	AGTTTTGCATGCAAGGGTGG	9
rs3219175	TCCTCCAGCCCTTACTGTCT	GCTTGGCTAATAAGTCCCTG	111
rs3219177	TCAATGAGAGGATCCAGGAG	TGTGCCAGGGATCAGTGAG	120
rs34861192	CAGTGCTGTGATCATAAGTC	TACTAAGGAGGCTGACGTG	96
rs1862513	AGACAGTAAGGGCTGGAGGA	ACCACCTCCTGACCAGTCT	107
rs3745367	ATTCAACCCCAACTCCACTC	GAAGGTTTGGAGTGAGAGCG	110
-180C/G	CACCACCTCCTGACCAGTCT	CACCACCTCCTGACCAGTCT	242
rs3745369	TAGCGTCTCCAAGAGAGTG	AGTTGGAGACCCCATAGGAG	120

Polymerase chain reaction (PCR): The whole reaction volume was 20 μl, consisting of 2 μl 1× buffer, 0.6 μl 3 mM Mg^2+^, 2 μl 2 mM dNTP, 2 μl forward primers and 2 μl reverse primers of genomic DNA, and 1 unit of Taq DNA polymerase. Procedures: (1) Pre-denaturalizing at 95? for 2 min; (2) Cycling for 40 cycles as 94? for 90 s, 53? for 90 s, and 65? for 30 s; (3) Heating at 65? for 10 min. After the reaction, we took 2 μl of the products out for an agarose gel electrophoresis assay in 3% agarose gel.

Ligase detection reaction (LDR): 1 μl 1× buffer, 2 pmol / μl of each probe, 4 μl of the PCR products, and 2 units of NEB Taq DNA ligase. Procedures: (1) Pre- denaturalizing in 95? for 2 min; (2) Cycling for 40 cycles at 94? for 15 s and 50? for 25 s. We took 1 μl of the LDR ligation products out for the mixture with 1 μl ABI GS-500 ROX internal lane standards and 1 μl deionized methanamine buffer. We heated the mixture at 95? for 2 min for denaturalization and then rapidly cooled the mixture in the ice water mixture.

Sequencing electrophoresis and analysis of LDR products: A 3000 V capillary electrophoresis was performed in 5 % polyacrylamide and 5 mol/L urea for 2.5 hours with a PRISM 3730 DNA Sequenator. GENESCAN™672 and Genemapper software were utilized for data collection and SNP classification.

DNA sequencing for the PCR products: We randomly selected two samples of the PCR products for the wild-type, homozygous and heterozygous variants of the 9 SNPs in the resistin gene. Shanghai Biowing Applied Biotechnology Co. Ltd was employed for sequencing the certifying works.

### Statistical analysis

General statistics were completed with SPSS Statistics Software (version 18.0). A normal distribution test and a homogeneity test of variance were applied for the comparison of measurement data. A t-test was used for comparing the measurement data between the two normally distributed groups, and a rank-sum test was used for the non-normally distributed data. A χ^2^ test was utilized for the genotype frequencies. Meanwhile, after revising the exposure factors such as age, gender, blood glucose, hypertension and hyperlipoidemia with a nonparametric test and dual logistic regression arithmetic, we calculated the odds ratio (*OR*) and 95% confidence interval to analyze the relationship between gene sites and CI in different genetic models. With SHEsis Software, *D’* (Lewomin coefficient) was computed after the linkage disequilibrium (LD). A strong LD was believed to exist when *D’*>0.7, and haplotype analysis was then required [32].

### RESULTS

#### General characteristics of the subjects in the middle-aged groups

In the middle-aged groups (aged 45-65), there were 366 subjects in the CI group and 268 in the control group. Compared with the subjects in the control group, morbidity of hypertension, systolic pressure, morbidity of diabetes and parameters for blood glucose and low density lipoprotein significantly increased (*P*=0.001, *P*=0.001, *P*=0.001, *P*=0.002 and *P*=0.035), while high density lipoprotein level decreased (*P*=0.001). However, no evident differences were observed in average age composition, triglyceride level, total cholesterol level and the occurrence of hyperlipoidemia (*P*>0.05) (Table 2).

**Table 2:** General data of CI patients and healthy control subjects (45-65)

Characteristic	Case	Control	*P*
Number	366	268	
Age	56.29 ± 5.28	55.77 ± 5.70	0.258
Men, No (%)	226 (62)	131 (49)	0.005*
Triglyceride, mM	1.96 ± 1.25	1.83 ± 1.34	0.071
Total cholesterol, mM	4.70 ± 1.18	4.69 ± 1.01	0.749
HDL-C, mM	1.12 ± 0.38	1.30 ± 1.38	0.001*
LDL-C, mM	3.01 ± 0.96	2.84 ± 0.80	0.035*
Glucose, mM	6.32 ± 2.73	5.53 ± 2.59	0.002*
SBP, mmHg	142 ± 20	132 ± 16	0.001*
DBP, mmHg	83 ± 11	80 ± 10	0.058
Diabetes, No (%)	127 (34.7)	53 (19.8)	0.001*
Hyperlipidemia, No (%)	215 (58.7)	138 (51.4)	0.069
Hypertension, No (%)	155 (42.3)	70 (26.1)	0.001*

CI: cerebral infarction; HDL-C: high-density lipoprotein cholesterol; LDL-C: low-density lipoprotein cholesterol; SBP: systolic pressure; DBP: diastolic pressure; mean ± standard deviation: assessed by *t*-test;%percent: assessed by Chi-square test;

* *P*-value: significant difference, *P*<0.05.

#### Genotype distribution of 9 resistin gene sites in CI group and control group in the middle-aged groups

The genotype frequency distribution of the 9 SNPs in the CI group and the control group accorded with the Hardy-Weinberg Law (*P*>0.05), which indicated that the selected population was representative enough to carry out the statistical analysis. Three genotypes were detected, wild-type, homozygous or heterozygous variants with LDR. In the population aged over 65, compared with the control group, no obvious differences in frequency distribution for the 9 sites (rs3745368, rs34124816, rs3219175, rs3219177, rs34861192, rs1862513, rs3745367, 180C/G and rs3745369) in the resistin gene were found in the CI group. However, in the middle-aged population (45-65), the number of the GG genotype carriers of rs3219175 and rs34861192 in the CI group were evidently higher than those in the control group, which were 76.0% vs 67.9% (*P*=0.025) and 75.7% vs 67.9% (*P*=0.031), respectively. Nevertheless, no significant conclusion was discovered for the other seven SNPs (Table 3).

#### Association of SNPs rs3219175 and rs34861192 with CI in genetic models in the middle-aged groups

To evaluate the relationship between the resistin gene polymorphism and the onset risks of CI, we utilized dual logistic regression analysis to establish dominant, recessive and superdominant models. The models indicated that with revised risk factors such as age, gender, hypertension and hyperglycemia, the relationship between CI and SNPs rs3219175 and rs34861192 were observed in the dominant and the superdominant models (*P*<0.05) (Table 4). In the dominant (GG VS AG+AA) model, the onset risk of CI for GG genotype carriers of rs3219175 and rs34861192 was 1.49 and 1.46 times as high as that in the total sample of AG and AA genotype carriers (95% CI:1.03-2.71, *P*=0.036;95% CI:1.01-2.13, *P*=0.046). In the superdominant model (AA+GG VS AG), the onset risk for the total sample of AA and AG genotype carriers was 1.60 times as high as that in AG carriers (95% CI:1.09-2.34, *P*=0.017;95% CI:1.07-2.28, *P*=0.022).

**Table 3:** Genotype frequency distribution of 9 resistin gene sites in CI group and control group (45-65)

SNP	Case	Control	*P*
rs3745368			
AA	9 (2.5)	7 (2.6)	
AG	94 (25.7)	68 (25.4)	
GG	263 (71.9)	193 (72.0)	0.990
rs34124816			
AA	293 (80.1)	224 (83)	
AC	68 (18.6)	41 (15.3)	
CC	5 (1.4)	3 (1.1)	0.527
rs3219175			
AA	8 (2.2)	3 (1.1)	
AG	80 (21.9)	83 (31.0)	
GG	278 (76.0)	182 (67.9)	0.025^*^
rs3219177			
CC	347 (94.8)	250 (93.3)	
CT	18 (4.9)	18 (6.7)	
TT	1 (0.3)	0 (0.0)	0.438
rs34861192			
AA	8 (2.2)	3 (1.1)	
AG	81 (22.1)	83 (31.0)	
GG	277 (75.7)	182 (67.9)	0.031^*^
rs1862513			
CC	42 (11.5)	30 (11.2)	
CG	161 (44.0)	113 (42.2)	
GG	163 (44.5)	125 (46.6)	0.869
rs3745367			
AA	48 (13.1)	34 (12.7)	
AG	178 (48.6)	130 (48.5)	
GG	140 (38.3)	104 (38.8)	0.983
180C/G			
CC	161 (44.0)	125 (46.6)	
CG	163 (44.5)	113 (42.2)	
GG	42 (11.5)	42 (11.5)	0.798
rs3745369			
CC	49 (13.4)	35 (13.1)	
CG	167 (45.6)	111 (41.4)	
GG	150 (41.0)	122 (45.5)	0.501

*P* value: assessed by SHESIS Software.

#### LD and haplotype analysis of resistin 9 sites in the middle-aged groups

**Figure 1.**
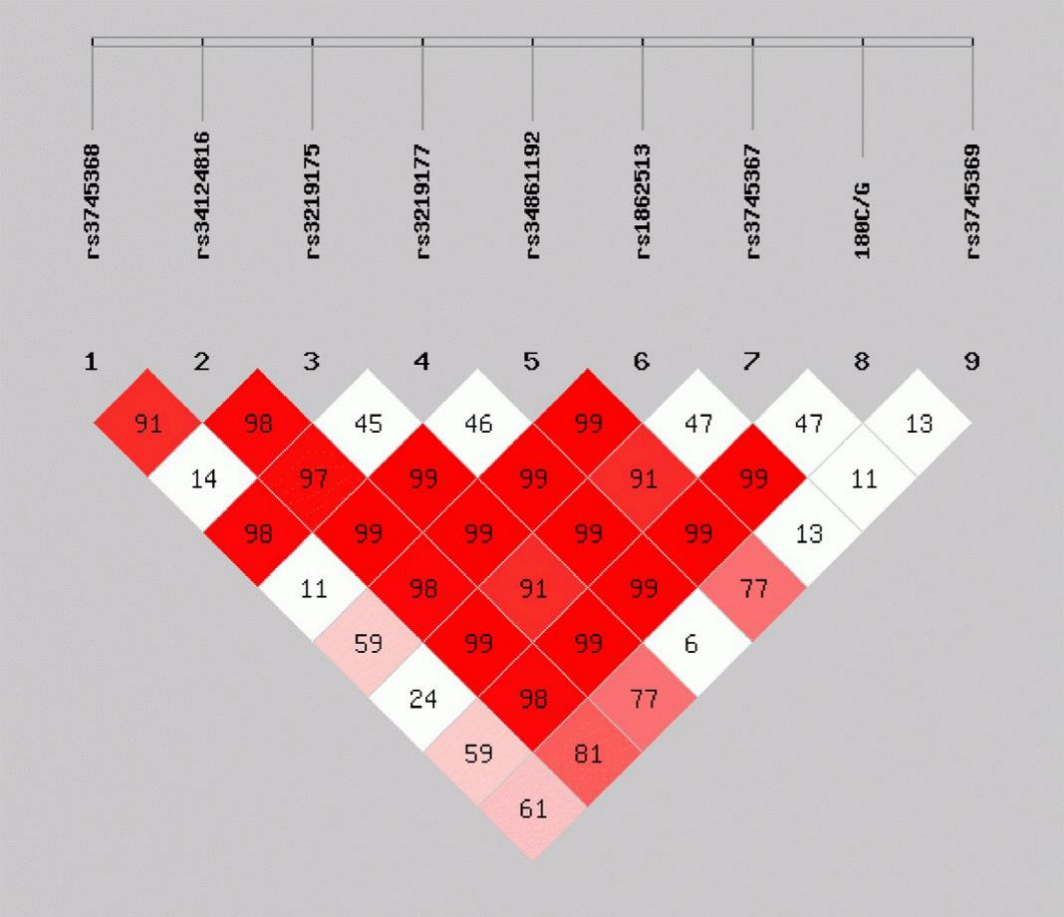
**LD analysis of 9 SNPs (subjects aged 45-65)**. The LD structure was analyzed by Haploview for a total of 634 alleles from the patients with cerebral infarction and from the controls. There was a strong LD between the rs34124816, rs3219175, rs34861192, rs1862513, rs3745367, 180C/G and rs3745369 (red box) polymorphisms with a standardized disequilibrium coefficient (*D’* >0.7).

As for the complex diseases caused by multi-genes, the function of single gene sites could be minute; thus, linkage analysis of multiple genes is necessary[33]. According to the LD coefficient *D’* among the 9 SNPs, strong LD existed among the seven SNPs (rs34124816, rs3219175, rs34861192, rs1862513, rs3745367, 180C/G and rs3745369) (*D’*>0.7) (Figure 1). Therefore, haplotype analysis needed to be performed. The haplotype of RETN SNPs rs34124816, rs3219175, rs34861192, rs1862513, rs3745367, 180C/G and rs3745369 (AGGCAGC) in the CI group had a 1.97 times higher occurrence rate than that in the control group (95% CI 1.07-3.60, *P*=0.026). In addition, the occurrence of CI also increased significantly. However, no evident or significant differences were observed between the CI group and the control group for the other 7 haplotypes (Table 5).

#### Genotype distribution of resistin gene sites rs3219175 and rs34861192 in the CI group and the control group with classification of gender and TOAST in the middle-aged groups

##### (1) The relationship between gender and rs3219175 and rs34861192

In middle-aged male subjects, the p-values for the genotype distribution of rs3219175, rs34861192 sites in the CI group and the control group were 0.021 and 0.028, respectively, which indicated statistical significance. As for middle-aged female subjects, the p-values were 0.729 and 0.729, indicating no statistical significance. Thus, CI induced by mutations of rs3219175 and rs34861192 could be related to gender and mainly impact the middle aged male CI patients (Table 6).

##### (2) The relationship between SAO, LAA and rs3219175, rs34861192

The p-value for the genotype distribution of rs3219175 and rs34861192 sites in the SAO CI group and the control group were 0.025 and 0.032, which indicated their statistical significance. As for the LAA CI group, the p-values were both higher than 0.05, indicating no statistical significance. Therefore, the mutations of the rs3219175 and rs34861192 SNPs mainly occurred in the SAO CI patients (Table 7).

**Table 4:** Relationship between CI and rs3219175, rs34861192 in different genetic models (45-65)

Locus	Genotype	Adjusted OR(95%CI)	*P* adjusted
rs3219175			
Dominant	AG+AA	1.00	
	GG	1.49 (1.03-2.17)	0.036^*^
Recessive	AA	1.00	
	AG+GG	0.55 (0.14-2.26)	0.407
Overdominant	AG	1.00	
	AA+GG	1.60 (1.09-2.34)	0.017^*^
rs34861192			
Dominant	AG+AA	1.00	
	GG	1.46 (1.01-2.13)	0.046^*^
Recessive	AA	1.00	
	AG+GG	0.55 (0.14-2.26)	0.407
Overdominant	AG	1.00	
	AA+GG	1.60 (1.07-2.28)	0.022^*^

*P* adjusted: assessed by logistic regression; adjusted for age, gender, hypertension, hyperlipidemia and diabetes mellitus.

**Table 5:** Association analysis of resistin haplotypes with CI (45-65)

Haplotype	Case (freq)	Control (freq)	*P* value	OR (95%CI)
AAACAGG	80.43 (0.11)	78.75 (0.15)	0.055	0.72 (0.52-1.01)
AGGCAGC	39.11 (0.05)	15.09 (0.02)	0.026^*^	1.97 (1.07-3.60)
AGGCAGG	30.81 (0.04)	19.30 (0.04)	0.566	1.19 (0.66-2.12)
AGGGACC	79.23 (0.11)	44.52 (0.08)	0.124	1.35 (0.92-1.99)
AGGGACG	34.52 (0.05)	31.66 (0.06)	0.361	0.79 (0.48-1.30)
AGGGGCC	69.88 (0.10)	69.62 (0.13)	0.058	0.71 (0.50-1.01)
AGGGGCG	300.27 (0.41)	217.20 (0.41)	0.772	1.04 (0.82-1.30)
CGGCGGC	67.90 (0.09)	41.78 (0.08)	0.332	1.22 (0.82-1.83)

*P* value: assessed by SHESIS Software. Note: the haplotypes whose frequencies in CI group and control group were both less than 5% were deleted.

### DISCUSSION

This study showed that the existence of the GG genotype of the two resistin gene promotor sites rs3219175 and rs34861192 could increase the occurrence of CI, while the other 7 SNPs were not significantly related to the occurrence. In the elder group (>65), no statistical significance was found between resistin gene polymorphism and CI. Because of the importance of age, gender, hypertension and hyperglycemia in CI onset [5], excluding these risk-factor deviations with dual logistic regression analysis, rs3219175 and rs34861192 appeared to be related to the occurrence of CI in the middle-aged patients in the dominant and superdominant models. Meanwhile, in the haplotype analysis, the haplotype of RETN sites rs34124816, rs3219175, rs34861192, rs1862513, rs3745367, 180C/G and rs3745369 (AGGCAGC) in the CI group had a 1.97 times higher occurrence rate than in the control group.

It has been reported that the polymorphism of rs34861192 and rs3745368 located on the resistin gene influenced the serum resistin level [34], which thereby influenced coronary artery calcification in a Japanese population [15, 35]. Thus the polymorphism of the resistin gene can be a basis for the prediction of coronary atherosclerotic cardiopathy. In addition, it is a remarkable fact that serum resistin level could impact the mortality and disability rate of CI patients over the 5 years after the onset of CI, which was discovered by Efstathiou [36]. Therefore, it is reasonable to assume that the polymorphism of the resistin gene may cast some effect on the incidence of CI. However, no clear report has been published about the relationship between CI and the polymorphism of the resistin gene until now.

**Table 6:** Genotype frequency distribution of rs3219175, rs34861192 in CI group and control group for different gender

SNP	Case(male)	Control(male)	*P*
rs3219175			
AA	5 (2.30)	2 (1.50)	
AG	44 (20.3)	44 (33.6)	
GG	168 (77.4)	85 (64.9)	0.021^*^
rs34861192			
AA	5 (2.30)	2 (1.50)	
AG	45 (20.7)	44 (33.6)	
GG	167 (77.0)	85 (64.9)	0.028^*^
SNP	Case (female)	Control (female)	
rs3219175			
AA	3 (1.70)	1 (0.70)	
AG	53 (29.6)	39 (28.5)	
GG	123 (68.7)	97 (70.8)	0.729
rs34861192			
AA	3 (1.70)	1 (0.70)	
AG	53 (29.6)	39 (28.5)	
GG	123 (68.7)	97 (70.8)	0.729

*P* value: assessed by SHESIS Software.

At the early stage, we selected 550 CI patients and 313 healthy control subjects. In a Chinese Han middle-aged population (ages 45-65), the genotypes of rs3219175 and rs34861192 were related to the susceptibility of CI, and this result was meaningful in various genetic models. However, no significant difference was observed between the CI group and the control group in the genotypes of the rs3745368, rs34124816, rs3219177, rs1862513, rs3745367, 180C/G and rs3745369 sites. As in Apalasamy’s research on the relationship between obesity and polymorphism of rs3219175, rs34861192 with serum resistin level in a Malaysian population, obesity was found to be related to gene polymorphism and serum resistin levels [37]. We assumed that the polymorphism of rs3219175 and rs34861192 could influence the serum resistin level and further impact the occurrence of CI. Nevertheless, this hypothesis required a large sample of the Chinese population for more data on resistin levels. What interested us was that, with classification according to gender and TOAST standards, in the middle-aged male subjects, the number of GG genotype carriers in the CI group was much higher than that in the control group with no statistical significance in the middle-aged female subjects. As reported by Sigri Beckers, in a sample of Belgian women, gene polymorphisms of rs1862513, rs3745367 and rs3745369 in the resistin gene were not related to obesity [38]. Thus gene polymorphisms of rs34861192 and rs3219175 might also not be related to the occurrence of CI in females. Additionally, according to TOAST, CI can be classified into 5 subtypes: LAA, CE, SAO, SDE SUE [27]. To decrease the impact from cardiac and other factors, we excluded CE, SDE and SUE patients. We found an obvious difference between the SAO subjects and the healthy control subjects in their genotype distributions of rs3219175 and rs34861192. However, when we turned to the LAA CI and the control subjects, no statistically significant difference was observed. In addition, as one of the most studied sites, the resistin promotor sequence rs1862513 was found to be related to a high resistin level and to type II diabetes in a Japanese sample [39],but merely resistin level in Korean [40]. We found no relationship between rs1862513 and CI in the Chinese Han population. Thus, the discovered functions of this site are different in different populations [41, 42], which indicates that, in different populations, the polymorphism of different resistin gene sites might possess different clinical significance for different diseases.

In the Chinese Han middle-aged population, a strong LD existed between resistin gene sites rs3219175 and rs34861192, and the impacts of their gene polymorphisms on CI appeared similar. As reported in a Japanese paper, a strong LD also existed between rs3219175 and rs34861192 in research on the relationship between resistin gene polymorphism and serum resistin level in a Japanese population [25], which accords with our results. However, in this study, they found that a nucleoprotein combined with the DNA sequence of rs3219175, but not of rs34861192, in the gel electrophoretic mobility shift assay, and the transcriptional factor binding sites also existed in rs3219175. Thus, this study speculated that rs3219175 plays the main role [25].

**Table 7:** Genotype frequency distribution of rs3219175, rs34861192 in CI group and control group for LAA and SAO

SNP	Case (SAO)	Control	*P*
rs3219175			
AA	7 (2.70)	3 (1.10)	
AG	55 (21.5)	83 (31.0)	
GG	194 (75.8)	182 (67.9)	0.025^*^
rs34861192			
AA	7 (2.70)	3 (1.10)	
AG	56 (21.9)	83 (31.0)	
GG	193 (75.4)	182 (67.9)	0.032^*^
SNP	Case (LAA)	Control	*P*
rs3219175			
AA	1 (0.10)	3 (1.10)	
AG	25 (22.9)	83 (31.0)	
GG	83 (76.1)	182 (67.9)	0.283
rs34861192			
AA	1 (0.10)	3 (1.10)	
AG	25 (22.9)	83 (31.0)	
GG	83 (76.1)	182 (67.9)	0.283

*P* value: assessed by SHESIS Software.

Some limitations certainly exist in our study. First, the sample was relatively small. Second, the selected subjects were all between 45 and 75 years old, which means our study was targeting mainly the susceptibility of CI in a middle-aged population. Thus, there were not enough elder control subjects, and the research on the elder group was therefore impacted. Third, when collecting the information on the ailing and healthy subjects, with the difficulty of and limitations on blood sample collecting, the serum resistin level was not detected. Thus, we were unable to judge the influence from the resistin gene polymorphism on the serum resistin level of the CI patients. In conclusion, the major discovery of the current study first discovered in the Chinese Han middle-aged population, the GG gene type carriers of the resistin gene sites rs3219175 and rs34861192 had a high risk for CI onset, especially in middle-aged male patients and SAO CI in all middle-aged patients. In middle-aged population, the occurrence rate of the haplotype AGGCAGC (rs34124816, rs3219175, rs34861192, rs1862513, rs3745367, 180C/G and rs3745369) was 1.97 times (*P*<0.05) higher in the patient group than in the control group. To further make the relationship between the gene bands, the middle-aged male CI patients, and the SAO middle aged CI patients clear, we need to understand the relevance of the functions of sites rs3219175 and rs34861192 for the population, and explain the similarity of the sites rs3219175 and rs34861192 in the population. The study of these functions will require large samples, multiple locations and multiple races of subjects. To sum up, our study laid the foundation for early prevention, personal treatment and research on the related genetic mechanisms of CI with clinical significance.
